# Supplementary material for: Combined uranium-series and electron spin resonance dating from the Pliocene fossil sites of Aves and Milo’s palaeocaves, Bolt’s Farm, Cradle of Humankind, South Africa
Source: PeerJ. 2024 Jun 28;12:e17478. doi: 10.7717/peerj.17478 (PMC11216204; doi:10.7717/peerj.17478)
Supplement: Supplemental Information 1 [file peerj-12-17478-s001.docx]

Supplementary Materials for

**Oldest combined US-ESR dates from the Pliocene fossil sites of Aves and Milo’s palaeocaves, Bolt’s Farm, Cradle of Humankind, South Africa**

Wenjing Yu, Andy I.R. Herries, Tara Edwards, Brian Armstrong, Renaud Joannes-Boyau

Correspondence to: [wenjing.yu@scu.edu.com](mailto:wenjing.yu@scu.edu.com)

**This PDF file includes:**

Supplementary Text

Figs. S1 to S2

3D models. S3 to S4

**Other Supplementary Materials for this manuscript include the following:**

**Supplementary text**

***Fitting program***

The selection of fitting program is crucial in D_E_ calculation because it contributes significantly to the D_E_ value. Different fitting programs of dose determination using ESR spectra have been developed, and different functions have been tested in studies (Grün and Brumby 1994, Grün 2000a, 2002, 2006a). In the research of Duval and Grün (2016), SSE is recommended for D_E_ less than 300Gy; and for DE over 1000Gy, samples will show flat DRCs, fitting with DSE is physically more correct (Duval et al. 2009, 2013). However, the SSE function offers the best fitting performance for our ESR growth curves (Figure S1). And previous research, e.g. Herries et al., (2018) has proved that SSE function can provide reliable data on old sites such as Drimolen in the Cradle of Humankind. A further proof has also been applied on AV-ESR-02, the D_E_ was increased from 2103 ± 294 Gy to 2785 ±386 Gy after irradiated 600Gy for the same sample (Figure S2), this fits the 600Gy difference with D_E_ when using SSE function. A further test was made to compare the sample ages between original US-ESR isotopic ratio and US-ESR isotopic ratio with a reducing factor of 100 to check the accuracy and validity of the geochronological data (Table S1).


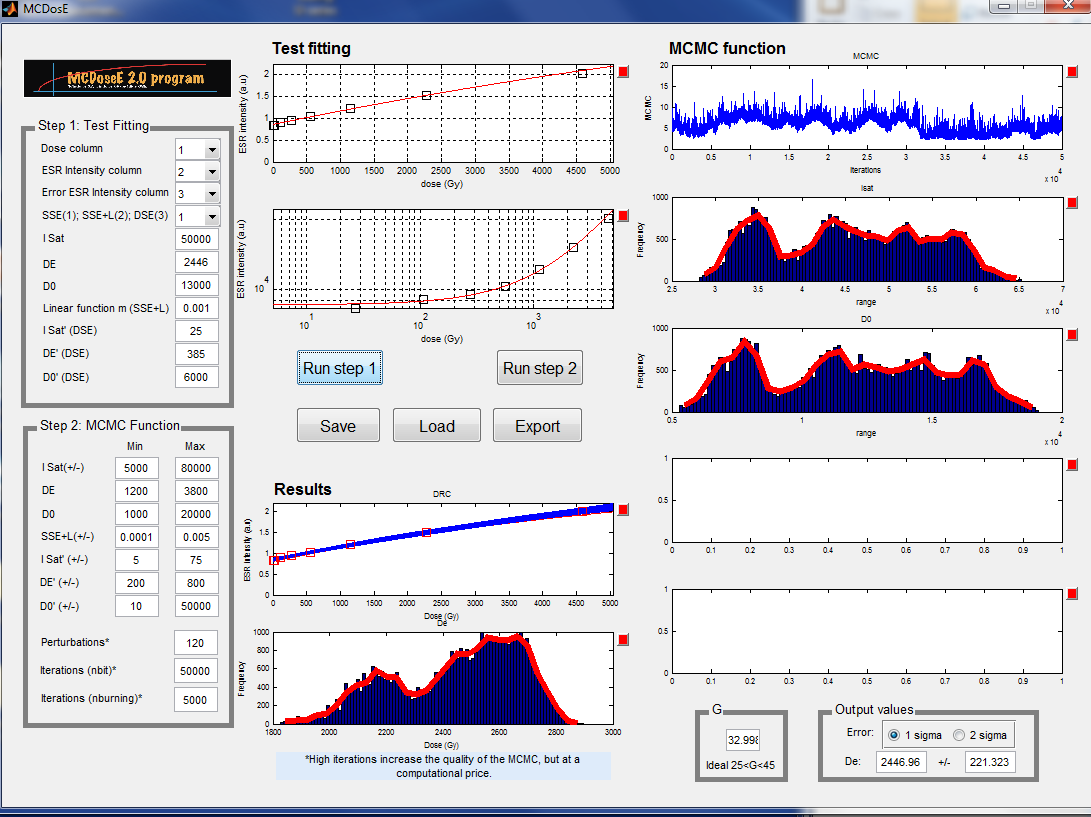


*Figure S1 Screenshot of the SSE fitting results of a fossil tooth sample (Sample ID: AV-ESR-02). SSE fittings in this research were conducted by McDoseE 2.0 fitting program.*


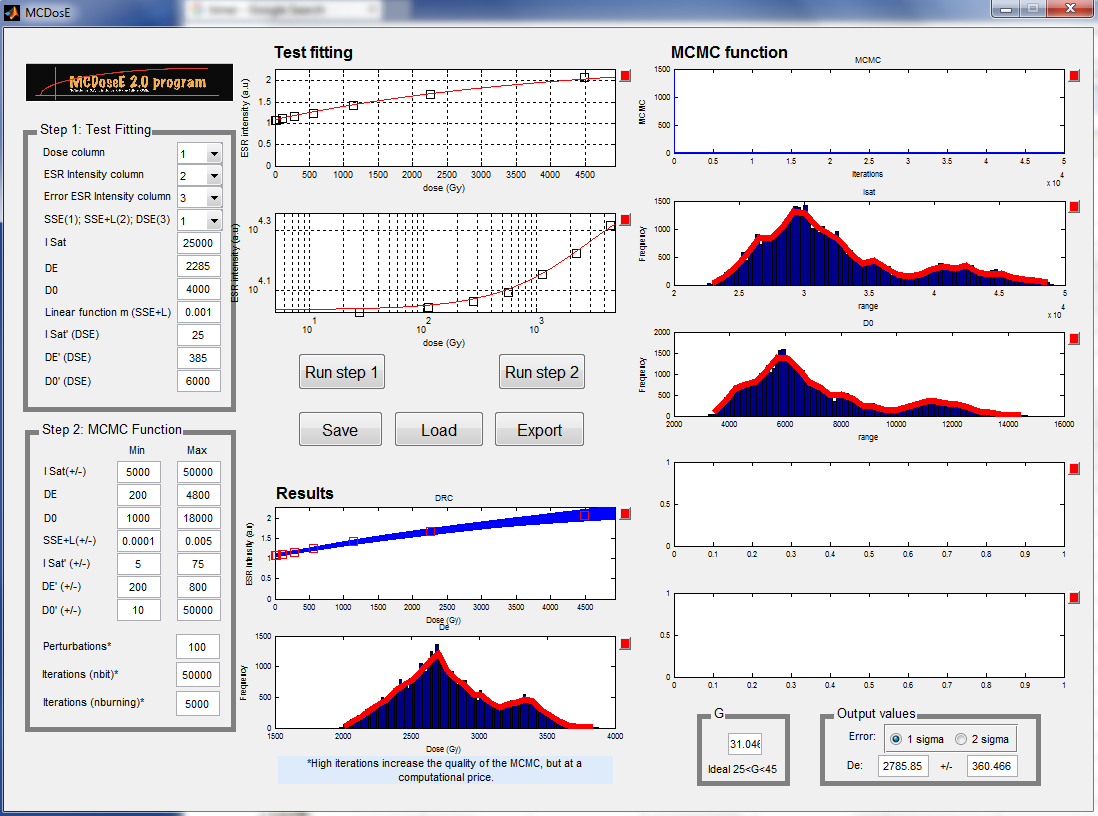


*Figure S2 Screenshot of the SSE fitting results of a fossil tooth sample (Sample ID:AV-ESR-01-600Gy) with pre-irradiated 600Gy to cross test the fitting program. SSE fittings in this research were conducted by McDoseE 2.0 fitting program.*

***3D models***

3D models for Aves cave and Milo’s Cave were created to show a better view of the sample locations and stratigraphy. Aves Cave sample locations of ESR samples (ESR 1-3), U-Pb samples (UPB03) and paleomagnetic samples (AV 08, AV 09 AV10, AV12) follow this link:

<https://sketchfab.com/3d-models/bolts-farm-aves-cave-874c2e1e996f44c9a723e85df53ee457>

| [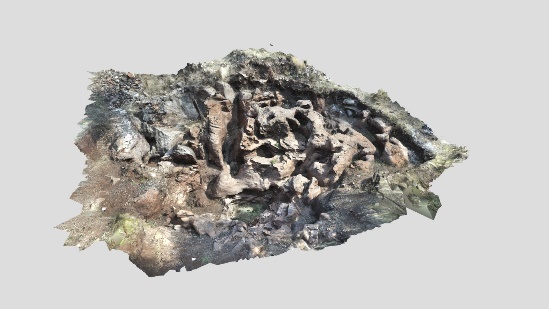](https://sketchfab.com/3d-models/bolts-farm-aves-cave-874c2e1e996f44c9a723e85df53ee457) | [Bolts Farm, Aves Cave - 3D model by b.armstrong](https://sketchfab.com/3d-models/bolts-farm-aves-cave-874c2e1e996f44c9a723e85df53ee457)  Aves cave sample locations and stratigraphy. Sample locations of ESR samples (ESR 1-3), U-Pb samples (UPB03) and paleomagnetic samples (AV 08, AV 09 AV10, AV12). 3D Model Created by Brian Armstrong December 2023 from photos collected in 2016 - Bolts Farm, Aves Cave - 3D model by b.armstrong  sketchfab.com |
| --- | --- |

Milos Cave sample locations of ESR samples follow this link:

<https://sketchfab.com/3d-models/bolts-farm-milo-3d-model-c5e076ce8dbc457c8d1f022e28f270ef>

| [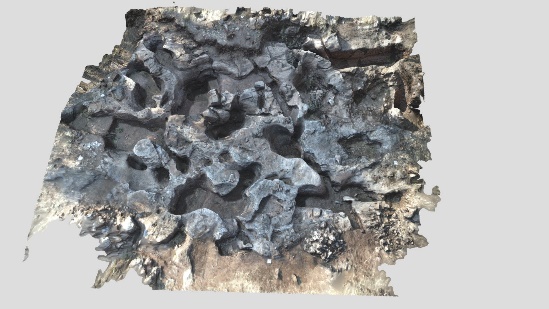](https://sketchfab.com/3d-models/bolts-farm-milo-3d-model-c5e076ce8dbc457c8d1f022e28f270ef) | [Bolts Farm Milo 3D Model - 3D model by b.armstrong](https://sketchfab.com/3d-models/bolts-farm-milo-3d-model-c5e076ce8dbc457c8d1f022e28f270ef)  Bolts Farm, Milo’s Cave showing the location of US-ESR samples 1-5. 3D Model Created by Brian Armstrong December 2023 from photos collected in 2016 - Bolts Farm Milo 3D Model - 3D model by b.armstrong  sketchfab.com |
| --- | --- |
