# Supplementary material for: Combined uranium-series and electron spin resonance dating from the Pliocene fossil sites of Aves and Milo’s palaeocaves, Bolt’s Farm, Cradle of Humankind, South Africa
Source: PeerJ. 2024 Jun 28;12:e17478. doi: 10.7717/peerj.17478 (PMC11216204; doi:10.7717/peerj.17478)
Supplement: Supplemental Information 2 — A state of equilibrium will be reached due to the highly mineralized teeth, Rn and Ra lose are thus not taken into account in the calculation. [file peerj-12-17478-s002.docx]

|  |  |  | **Fragments** |  | **Error** | **Powder** |  | **Error** | **Comment** |
| --- | --- | --- | --- | --- | --- | --- | --- | --- | --- |
| Ave's Cave | AV-ESR-01 | **USESR** | 3264 | ± | 642 | 2915 | ± | 402 |  |
|  |  | **USESR-no error** | 2971 | ± | 513 | 2716 | ± | 351 |  |
|  | AV-ESR-02 | **USESR** | ND | | | 2322 | ± | 357 | **Problematic** |
|  |  | **USESR-no error** | 3970 | ± | 640 | 2333 | ± | 370 |  |
|  | AV-ESR-03 | **USESR** | ND | | | 2759 | ± | 498 |  |
|  |  | **USESR-no error** |  |  |  | 2767 | ± | 468 |  |
| Milo's Cave A | MA-ESR-02 | **USESR** | 2954 | ± | 240 | 2285 | ± | 195 |  |
|  |  | **USESR-no error** | 2745 | ± | 458 | 2532 | ± | 344 |  |
|  | MA-ESR-03 | **USESR** | ND | | | NP | | |  |
|  | MA-ESR-03 | **USESR-no error** | ND | | |  |  |  |  |
|  | MA-ESR-04 | **USESR** | 2736 | ± | 248 | 2792 | ± | 202 | **Problematic** |
|  |  | **USESR-no error** | 2290 | ± | 358 | 2767 | ± | 451 |  |
|  | MA-ESR-05 | **USESR** | 2102 | ± | 267 | NP | | |  |
|  |  | **USESR-no error** | 1914 | ± | 258 |  |  |  |  |
